# Maladaptive secondary sexual characteristics reduce the reproductive success of hybrids between native and non‐native salmonids

**DOI:** 10.1002/ece3.4676

**Published:** 2018-11-14

**Authors:** Sho Fukui, Shannan L. May‐McNally, Eric B. Taylor, Itsuro Koizumi

**Affiliations:** ^1^ Graduate School of Environmental Earth Science Hokkaido University Sapporo Japan; ^2^ Department of Zoology, Biodiversity Research Centre and Beaty Biodiversity Museum University of British Columbia Vancouver British Columbia Canada; ^3^ Faculty of Environmental Earth Science Hokkaido University Sapporo Japan

**Keywords:** adaptive introgression, extrinsic factor, invasive introgression, invasive species, postzygotic isolation, sexual selection

## Abstract

Human‐mediated hybridization between introduced and native species is one of the most serious threats to native taxa. Although field studies have attempted to quantify the relative fitness or reproductive success of parental species and their hybrids**,** only a few studies have unraveled the factors determining the fitness of hybrids. Here, we hypothesized that maladaptive secondary sexual characteristics may reduce fitness of hybrids between two fish species. To test this, we evaluated the reproductive success of introduced brook trout (BT: *Salvelinus fontinalis*), native white‐spotted charr (WSC: *S. leucomaenis*) and their hybrids in a natural stream in Hokkaido, Japan, where the two parental species show remarkably different male secondary sexual characteristics, such as elongated jaws and deeper bodies. We predicted that introgression from WSC is maladaptive for BT males because the BT male has more prominent secondary sexual characteristics. Our results suggest that both sexual selection and outbreeding depression in males and females significantly influence an individual's reproductive success. Our results also suggest that asymmetric introgression may increase the risks to persistence in the recipient species.

## 1 INTRODUCTION

Human‐mediated hybridization has contributed to the decline and extinction of many populations and species of plants and animals (Allendorf, Leary, Spruell, & Wenburg, 2001; Rhymer & Simberloff, 1996). Introgressive hybridization of native species with non‐native species can cause the breakdown of inherent gene complexes and ecological adaptations in native populations, which can increase threats to the persistence of rare or endangered species (Rhymer & Simberloff, 1996). The most informative approach for estimating such effects of hybridization is to measure the relative difference in fitness between hybrids and parental species under natural conditions (Svedin, Wiley, Veen, Gustafsson, & Qvarnström, 2008). As the extent of hybridization and introgression can further be exacerbated by changes in land use and global climate change (Allendorf et al., 2001; Muhlfeld et al., 2014), there is a growing need for the suitable conservation planning of native species to better characterize how ecological factors involved in hybridization interact to influence the spread or existence of non‐native genes within and among native populations.

Compared to extensive studies on natural hybrid zones (Hochkirch & Lemke, 2011; Lemmon & Lemmon, 2010; Lowry, Modliszewski, Wright, Wu, & Willis, 2008), ecological factors affecting hybrid fitness have been much less well investigated in human‐mediated hybridization (but see, While, Michaelides, & Heathcote, 2015). Since it is generally difficult to track hybrid individuals simultaneously with their parental species in the wild while measuring fitness component(s), several previous studies have illustrated the patterns or consequences of hybrid fitness in the wild (Muhlfeld et al., 2009; Taylor, Gerlinsky, Farrell, & Gow, 2012), but less frequently have the ecological mechanisms driving such patterns or consequences been studied. As an outstanding example, flycatcher birds from a natural hybrid zone have been well investigated for factors such as hybrid inviability (Alatalo, Gustafsson, & Lundberg, 1982), hybrid infertility (Alund, Immler, Rice, & Qvarnstrom, 2013), ecological inviability (Tegelstrom & Gelter, 1990; Veen et al., 2001), and sexual selection against hybrids (Svedin et al., 2008). Understanding the factors influencing hybrid fitness is critical for forecasting the impacts of human‐mediated hybridization between native and non‐native species (Allendorf et al., 2001; Ellstrand, Prentice, & Hancock, 1999; Fitzpatrick et al., 2010).

Sexual selection can be a key factor influencing hybrid fitness (Lemmon & Lemmon, 2010; Svedin et al., 2008; While et al., 2015), but has been studied much less than other postzygotic factors, such as genetic incompatibilities (Brown & Eady, 2001; Howard, Gregory, Chu, & Cain, 1998; Noor, 1997), or hybrids performing poorly in parental niches (Grant & Grant, 1997; Hatfield & Schluter, 1999; Naisbit, Jigginns, & Mallet, 2001). A study by Svedin et al. (2008) estimated the fitness components of collared (*Ficedula albicollis*) and pied (*Ficedula hypoleuca*) flycatchers and their hybrids and found that sexual selection against the intermediate phenotype (i.e., intermediate plumage characters) of hybrid males accounted for approximately one‐third of the total postzygotic isolation between the two species, and approximately 75% of this selection was attributable to sexual selection. Despite the potential importance of sexual selection, its role in postzygotic isolation has only been investigated in a few other cases, such as acoustic signals of the chorus frog (*Pseudacris* spp, Lemmon & Lemmon, 2010) and the body colors and head length of the wall lizard (*Podarcis muralis*, While et al., 2015).

**Figure 1:**
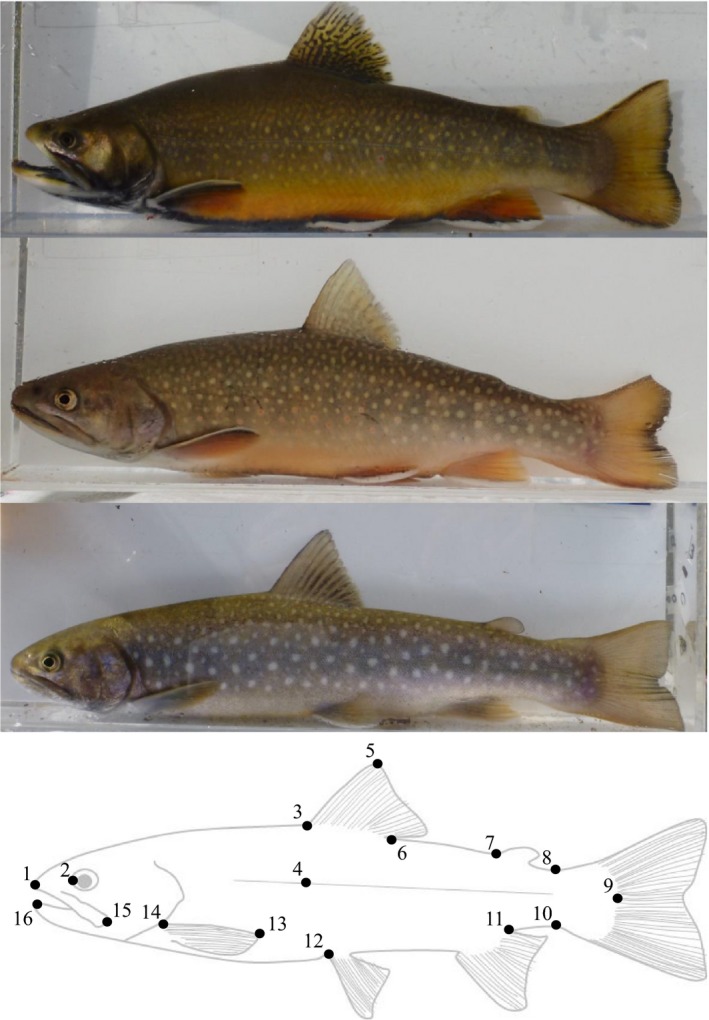
Representative males of introduced brook trout (above photo), hybrid (middle photo), and native white‐spotted charr (below photo). Photographs have been taken in the present study area. Bottom drawing picture represents locations for 16 landmarks identified on each male charr photograph:snout length (1–2), under‐jaw length (15–16), pectoral fin (13–14), dorsal fin height (3–5), hump height 1 (3–4), hump height 2 (6–12), caudal peduncle (8–10), caudal length 1 (7–9), caudal length 2 (9–11). Theses nine measurements are used in the principal component analysis. For females, only caudal peduncle (8–10) was used for subsequent statistical analysis with the zero‐inflated model

In this study, we explored the effects of sexual selection against hybrids between native and non‐native salmonid species (i.e., charr: *Salvelinus* genus). We focused on secondary sexual characteristics of hybrid males because male‐male competition often results in remarkable sexual dimorphisms in salmonid fishes (Fleming, 1996; Fleming & Gross, 1994; Quinn & Foote, 1994). During the breeding season, males develop elongated jaws, develop an angular tip or kype on the lower jaw, and large canine‐like teeth emerge from the hooked snout which appear to function as specialized weapons in fighting competitors. In addition, the enlargement of the dorsal hump appears to act as a shield against attack from competitors and block access to ovipositing females by competing males. Males also show brilliant nuptial coloration, which may attract females. Such developed secondary sexual characteristics can contribute to reproductive success through male‐male competition and female choice (Fleming & Gross, 1994; Quinn & Foote, 1994). Hybrids between introduced brook trout (BT), *Salvelinus fontinalis*, with native white‐spotted charr (WSC), *S. leucomaenis*, occur in a boreal stream of Japan (Fukui, May‐McNally, Katahira, Kitano, & Koizumi, 2016; Kitano, Ohdachi, Koizumi, & Hasegawa, 2014). Here, the male secondary sexual characteristics between these species express obviously distinct features; BT has more intense breeding coloration, deeper bodies, and larger jaws (Figure 1, see Results). These observations allowed us to test whether the male reproductive characters can be one of the factors/mechanisms determining the hybrid fitness in the wild. For a similar and well‐investigated example, introgression between non‐native rainbow trout (*Oncorhynchus mykiss*) and native westslope cutthroat trout (*O. clarkii lewisi*) has spread across watersheds in North America (Boyer, Muhlfeld, & Allendorf, 2008; Hitt, Frissell, Muhlfeld, & Allendorf, 2003; Yau & Taylor, 2014). Muhlfeld et al. (2009) found that the reproductive success in post F1 hybrids declined rapidly with an increasing genetic proportion of rainbow trout ancestry, although the mechanisms reducing the reproductive success of hybrids remained unknown. Here, we conducted an intensive field survey with parentage analysis in a tributary of the upper Sorachi River, Hokkaido, Japan, to (a) quantify the reproductive success of parental species and their hybrids in a wild population, (b) evaluate whether introgression causes outbreeding depression in the life stages from maturation to the juveniles of the next generation, and (c) test whether male reproductive characters affects hybrid fitness. For (b) and (c) we focused on BT and its hybrid because sample size was limited for pure WSC. We specifically predicted that transfer of the less well developed secondary sexual characteristics of native WSC would reduce the male reproductive success of introduced BT.

## 2 MATERIALS AND METHODS

### 2.1 Study area and data collection from the field

Our field study was conducted in the Higashi‐Furebetsu stream, a tributary of the upper Nunobe stream, in central Hokkaido, Japan (Supporting Information Figure S1). Based on species distribution data from a previous survey (Fukui et al., 2016), we considered that the tributary was suitable to investigate the interactions between WSC and BT because both species and their hybrids were consistently observed in the tributary and the tributary size was relatively small (2–4 m in stream width). Fourteen contiguous sections from a 2,615 m length stream reach were used as permanent study sites with a length of 100–425 m (mean = 186.8 m) in each section. Other species collected during the field survey included freshwater sculpin (*Cottus nozawae*) and Siberian stone loach (*Barbatula barbatula*).

The field survey in the Higashi‐Furebetsu stream was conducted in August, October, and December 2013, May, June, July, and October 2014, and July 2015. The survey was mainly concentrated on collecting adult individuals in 2013, age 0 + juveniles in 2014 and age 1 + juveniles in 2015 to conduct a subsequent parentage analysis for reproductive success. This sampling design evaluated detailed reproductive success of 2013‐year adult fish by collecting their offspring (i.e., 2014‐year cohort). We passed through each section two to four times using backpack electrofishing (200–300 V). Significant effort was made to collect all of the potential parents and their offspring inhabiting this study area to estimate reproductive success. Fish captured were anesthetized and measured for fork length (FL: nearest 1 mm), as an indicator of body size. The mean fork length (±*SD*, range, sample size) of mature BT, WSC, and hybrid (HYB) males successfully genotyped were 181.9 ± 34.4 mm (134–290 mm, *n* = 74), 213.6 ± 25.6 mm (162–265 mm, *n* = 16), and 189.2 ± 50.4 mm (129–323 mm, *n* = 22), respectively. Additionally, individuals captured in 2013 that exceeded 45 mm in FL were implanted with 8 or 12 mm passive integrated transponder (PIT) tags for individual identification. We implanted 1,095 individuals with PIT tags in 2013 and 2014. Fin clips of all candidate parental and offspring fish were preserved in 99% ethanol for subsequent DNA analysis. Fin tissues were stored at −20°C until DNA extraction.

Photographs were taken for all captured age–1+ and older fishes to measure the external characteristics of all candidate parental fish using an acrylic water tank (40 cm × 15 cm × 10 cm) and digital camera (Panasonic; DMC‐FT3‐S). Nine morphological characters (Figure 1) were measured from the photographic data using the software Image J (version 1.48v; US National Institutes of Health, Bethesda, Maryland) corrected by the fork length measured during the field sampling (i.e., calculated by relative lengths). Morphological differences between the three male groups (i.e., BT, HYB, and WSC) were examined by principal component analysis (PCA). All the nine characters measured were ln‐transformed and size‐adjusted using their FL prior to the PCA. Since the first principal component (PC1) included secondary sexual characters such as snout length or hump height, which are known as sexually selected variables (Fleming & Gross, 1994; Quinn & Foote, 1994), and showed a significant difference between WSC and BT (see Results), PC1 was used as a value of secondary sexual characteristics in the subsequent statistical analysis.

### 2.2 DNA analyses

Total genomic DNA was extracted from fin tissues using a PureGene DNA isolation kit (Applied Biosystems) following the manufacturer's protocol. The DNA was dissolved in 50 µl TE buffer. We used 13 microsatellite loci (Ssa197, Sle6, Sco200, SsosL456, Otsg83b, Smm21, Sco211, Sco216, Otsg253b, Sco220, MST‐85, Sfo12, and Omm1105) for parentage analysis. The loci Sfo12, Ssa197, and MST‐85 were known to be diagnostic markers to identify BT, WSC, and HYB (Kitano et al., 2014). Furthermore, allele sizes of SsosL456, Smm21, Otsg83b, Otsg253b did not overlap between BT and WSC (Supporting Information Table S1), when we used individuals which have ≥99.9% proportion WSC ancestry and ≥99.9% proportion BT ancestry subsequently calculated in STRUCTURE (Pritchard, Stephens, & Donnelly, 2000). Therefore, seven of the 13 loci had non‐overlapping (diagnostic) sets of alleles in BT and WSC. The PCR reactions were performed in 10‐µl volumes and the reaction mixtures contained 0.5 U Master Mix (GoTaq, Promega), 0.2 µM of each primer, 0.2 mM dNTP, 50 mM KCL, 15 mM Tris‐HCl (pH 8.0), 1.5 mM MgCl_2_, and about 50–100 ng/µl of genomic DNA as a template. The PCR themocycling protocol was as follows: 2 min at 95°C, followed by 35 cycles of denaturation at 95°C for 30 s, annealing at 56°C for 30 s, and extension at 72°C for 30 s. The amplified products were analyzed on the genetic analyzer ABI 3,130 (Applied BioSystems) and allele sizes were scored using GeneMapper (GeneMapper v.4.0; Applied BioSystems).

### 2.3 Data analysis

Parental species and hybrids were genetically determined using the software STRUCTURE (Pritchard et al., 2000). When all samples (i.e., BT, WSC, and HYB) were analyzed, the most likely number of putative populations (*K*) was two, representing WSC and BT. Based on *K* = 2, we calculated a hybrid index as the proportion WSC ancestry: this ranges from 0.001 for pure BT (no WSC allele) to 0.999 for pure WSC (no BT allele). First generation hybrids between BT and WSC have a hybrid index of 0.5 with the 13 microsatellite loci and heterozygous for alleles from each of the parental taxa at all the seven diagnostic loci (Supporting Information Table S1). We assumed that fish with a hybrid index of 0.5 but not heterozygous at all the diagnostic loci were post‐F_1_ hybrids, although it was difficult to distinguish backcrossing hybrids from other post‐F1 hybrids.

We performed parentage analysis using CERVUS v3.0.7 (Kalinowski, Taper, & Marshall, 2007) to identify parentage among candidate parents (the adults captured in October and December of 2013) and offspring (the 0+ fry sampled in May, June, August, October of 2014, and 1+ parr sampled in September 2015). The CERVUS analysis uses a maximum‐likelihood approach to predict parent‐offspring relationships. For each offspring, a metric of the likelihood that each potential father is the actual father is first calculated as a log‐likelihood (LOD) score. Then, CERVUS uses the range of LOD score distributions to calculate a critical LOD score against which LOD scores from the actual population can be evaluated. The LOD scores are then computed for each potential father using their actual genotype for each individual offspring. The critical LOD score calculated from the simulation step is used to assign fathers to each offspring (Kalinowski et al., 2007). The same procedure was also applied to mother‐offspring relationships. Allele frequencies using all genotyped individuals, the expected frequency of heterozygotes (*H*_e_), and the observed frequency of heterozygotes (*H*_o_) were also calculated using CERVUS.

We defined reproductive success as the number of offspring produced by each male or female. To investigate how body size, proportion WSC ancestry, and secondary sexual characteristics influence reproductive success, we used zero‐inflated models with negative binomial distribution (ZIB) to model counts of offspring (Zuur, Ieno, Walker, Saveliev, & Smith, 2009), including zeros for males that may have mated, but their offspring was not sampled, because the reproductive success, is a count variable with many zeros (Supporting Information Figure S2). The statistical analysis with ZIB was only conducted for BT and HYB because the distributions of reproductive success versus proportion WSC ancestry did not fit to the ZIB for WSC, likely attributable to the low sample size of pure WSC. The number of offspring was used as a response variable, whereas FL (i.e., body size: both males and females), PC1 (i.e., secondary sexual characters: males only), and the height of the caudal peduncle (females only) were used as independent variables. Because body size in salmonid fishes is important not only for male‐male interactions during mating but also for the establishment of territories in non‐breeding season (e.g., Fleming, 1996), we used both FL and PC1 as the predictor variables to separate these effects. The height of the caudal peduncle in females is associated with the depth of eggs buried and, thus, has a significant effect on reproductive success (Fleming & Gross, 1994). Since a significant correlation was observed between FL and PC1 (Spearman's *r* = 0.646, *p* < 0.001), PC1 was expressed as the residuals from its liner regression against FL. The transformation of PC1 value individuals with remarkable secondary sexual characteristics, such as higher hump height than average for their size, had a large positive residual value. All statistical analyses were performed using the software R version 3.2.4 (R Development Core Team, 2011).

## 3 RESULTS

In total, 666 offspring and 268 potential parents (149 females, 119 males) were collected in October and December 2013, May, June, July, and October 2014 and July 2015. Because the total of 934 samples included individuals with multiple captures, data on 61 recaptured individuals were removed from subsequent analysis. Of the 873 samples, 637 offspring and 236 mature individuals (124 females, 112 males) were successfully genotyped at a minimum of seven loci (i.e., 94.9% of possible genotypes). Of the 236 successfully genotyped mature individuals (i.e., the adults), 176 were pure BT (102 females, 74 males), 22 were pure WSC (6 females, 16 males), and 38 were putative hybrids (16 females, 22 males). The number of alleles per locus ranged from two to 24 (mean = 12.2), and expected heterozygosity ranged from 0.15 to 0.76 (mean = 0.41). This led to high combined exclusion probability for the whole set of loci in the parentage analysis (*Pe* > 0.85 for first parent and >0.99 for identity, Supporting Information Table S1). There were a number of loci that were not in Hardy–Weinberg equilibrium. This is likely because random mating did not occur among BT, WSC, and HYB.

**Figure 2:**
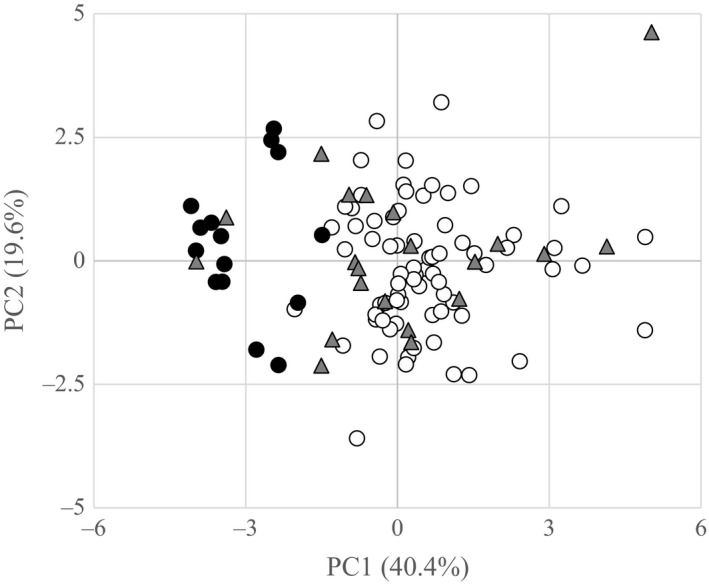
Principal component analysis incorporating nine morphological characters (snout length, under‐jaw length, pectoral fin, dorsal fin height, hump height 1, hump height 2, caudal peduncle, caudal length 1, caudal length 2) of the three male groups (native white‐spotted charr males, solid circles; hybrids males, gray triangles; introduced brook trout, open circles)

We analyzed morphological variation in males and we confirmed differences of secondary sexual characters between BT and WSC males. A total of 109 of the 112 males were successfully measured the morphological characters. Analysis of variance (ANOVA) indicated that these groups differed significantly from each other along the first principal component (PC1, *F*_2,107_ = 27.951, *p* < 0.0001). There was no significant difference among male types in PC2. The BT and WSC males were clearly distinguished by the secondary sexual characteristics (Figures 1 and 2, Table 1). Tukey's pairwise comparisons indicated that BT and WSC males significantly differed from one another in PC1 (*p* < 0.0001), and that HYB and WSC males significantly differed from one another in PC1 (*p* < 0.0001), but that BT and HYB groups were not significant distinct from one another (*p* = 0.4322). The PC1 is based primarily on variation in secondary sexual characters such as hump height and lower jaw length (Figure 1, Table 1) and explained 40.4% of the variation and PC2 explained an additional 19.6% of the variation.

**Table 1:** Eigenvector of morphological variables and the first two components (PC1 and PC2) obtained in the principal component analysis (PC1 explains 40.4% of the variation and PC2 an additional 19.6%)

Measurement	Variable	PC1	PC2
1–2	Snout length	0.34444	0.36449
15–16	Under‐jaw length	0.42814	0.26132
13–14	Pectoral fin length	0.22244	0.09270
3–5	Dorsal fin height	−0.08630	0.24612
3–4	Hump height 1	0.46144	0.01037
6–12	Hump height 2	0.46271	0.02523
8–10	Caudal peduncle	0.39417	−0.13014
7–9	Caudal length 1	0.14854	−0.61562
9–11	Caudal length 2	0.19153	−0.57708

Of the 637 offspring genotyped, 326 individuals (51%) were assigned to a sampled father, and 367 (58%) were assigned to a sampled mother by CERVUS. Of the 112 mature males, 59 (53%) appeared to produce at least one offspring (maximum 82 offspring) with the median of 1. Of the 124 mature females, 89 (72%) appeared to produce at least one offspring (maximum 84 offspring) with the median of 2. The reproductive success was a count variable with many zeros as expected (Supporting Information Figure S2).

**Figure 3:**
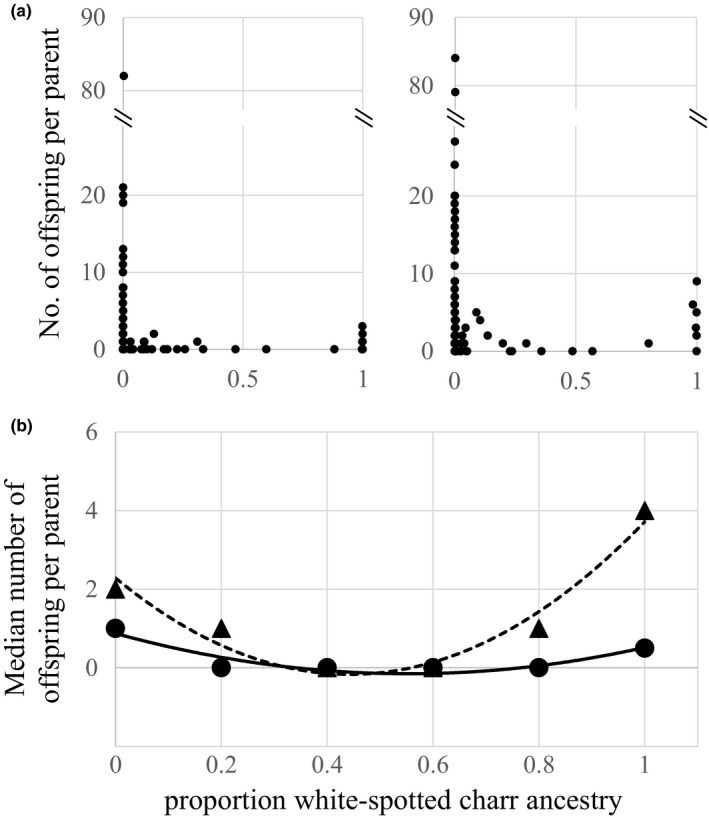
(a) Number of offspring per male (upper left) and female (upper right) versus the proportion native white‐spotted charr ancestry (i.e., “0” side is pure BT and “1” is pure WSC). The left plot includes 112 fathers and 327 juvenile assignments, and the right plot includes 124 mothers and 367 juvenile assignments using parentage analysis. Each point represents an estimate for an individual fish from a spawn year. Circles represent brook trout, white‐spotted charr, and their hybrids. (b) Plot of the median number of offspring per male (below: solid circles) and female (below: solid triangles) plotted against the proportion white‐spotted charr ancestry

**Figure 4:**
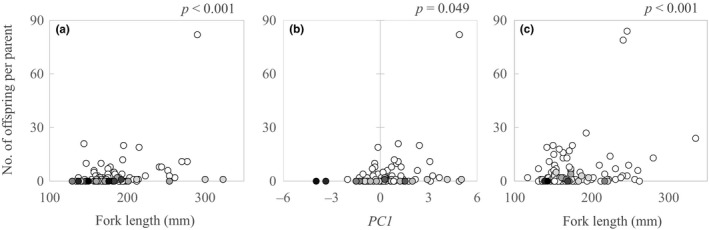
Reproductive success (number of offspring assigned per individual) as a function of male body size (a) and PC1 (b), and female body size (c) for brook trout and hybrid spawners. The *p*‐values of above predictor variables which were calculated in the zero‐inflated models (see Table 2.) are represented in the top of each plot figure. Open circles are individuals with more than 97% proportion brook trout ancestry, and graduated gray colors indicate proportion white‐spotted charr ancestry

The ZIB showed that the proportion of WSC ancestry among mature males and females had a strong negative effect on reproductive success (Table 2). Hybrids had lower reproductive success than that of both parental species in males and females (Figure 3a). Quadratic equations fitted to the median number of offspring per parent among males and females (males, *γ* ^2^ = 0.8673, *y* = 3.3482*x* ^2 ^– 3.7054*x* + 0.875; females, *γ* ^2^ = 0.9496, *y* = 12.5*x* ^2^ – 11.071*x* + 2.2857) estimated that reproductive success decreased by 30.6% and 25.0% for males and females, respectively, in BT with 20 percent proportion WSC ancestry (Figure 3b). For male BT and HYB, FL had only a small, but statistically significant positive effect on reproductive success (Table 2, Figure 4), but variation along PC1 (i.e., prominence of secondary sexual characters) had a 20*x* stronger positive effect on reproductive success (Table 2, Figure 4). For female BT and HYB, FL had a significant and positive effect on reproductive success, but caudal peduncle depth had no detectable effect on reproductive success (Table 2, Figure 4).

**Table 2:** Zero‐inflated count model with negative binomial distribution of male and female reproductive success

Factor	Coefficient	*SE*	*z*‐Value	*p*‐Value
Male model				
Intercept	−1.5238	0.8689	−1.7540	0.0795
Fork length	0.0147	0.0042	3.4600	0.0005
Proportion white‐spotted charr ancestry	−13.6403	5.1715	−2.6380	0.0084
PC1 (prominence of secondary sexual characters)	0.3073	0.1561	1.9680	0.0490
Female model				
Intercept	−10.6836	3.6995	−2.8880	0.0039
Fork length	5.4987	1.6464	3.3400	0.0008
Proportion white‐spotted charr ancestry	−9.8868	3.4380	−2.8760	0.0040
Caudal peduncle	−5.1735	6.9735	−0.7420	0.4582

The statistical analysis was conducted separately for males and females.

## 4 DISCUSSION

While past studies have shown the reduction in fitness or reproductive success in hybrids (e.g., Muhlfeld et al., 2009; Lancaster, Bradshaw, Goldsworthy, & Sunnucks, 2007), underling mechanisms have been rarely clarified (but see Svedin et al., 2008; Lemmon & Lemmon, 2010; While et al., 2015). We hypothesized that intermediate morphology is maladaptive especially the one related to sexual selection. Our results suggested that PC1 of morphological traits, which was associated with male secondary sexual characteristics, significantly affect the reproductive success of non‐native salmonid and its hybrids with native species. Importantly, hybrid index was also selected as a significant factor affecting male reproductive success, suggesting that not only maladaptive morphology but also some other undetermined factor(s) reduce the fitness of hybrids. Hybrid index was also negatively associated with female reproductive success. Although the sample size and number of examined loci were limited, our results were largely consistent with the predictions derived from the general patterns of salmonids’ mating systems. In addition, more prominent secondary sexual characteristics of non‐native species might result in asymmetric introgression from non‐native to native species, which is a serious concern from conservation perspective.

### 4.1 Roles of morphology on reproductive success of salmonids

Because of remarkable morphological variations during breeding seasons, many studies have attempted to clarify the factors affecting reproductive success in salmonids (e.g., Fleming & Gross, 1994; Tentelier et al., 2016). Among them, body size appeared to be the most consistent determinant as the variation could range from <10 cm to>100 cm, even within populations (L'Abbe‐Lund, 1989). Our population was less variable but still had more than twofold difference in fork length (129–323 mm). In males, large individuals become dominant and outcompete small males for access to females (Blanchfield, Ridgway, & Wilson, 2003; Dickerson, Brinck, Willson, Bentzen, & Quinn, 2005; Fleming, 1996, 1998 ; Quinn & Foote, 1994). In females, fecundity increases exponentially with body size and, thus, large females have much greater advantages (van den Berghe and Gross, 1989). While we found a significant effect of body size in both sexes, the size advantage was not very strong especially for males (Table 2; Figure 4). It is likely that small males use alternative tactics, such as sneaking, in the presence of bigger males to increase their reproductive success (Thériault et al., 2007). Alternatively, we might have missed some potential parents, which may weaken the pattern (see Limitations of the study section ). Importantly, however, although the body size is considered as the most promising factor, field studies under natural conditions often found little or no relationship between male size and reproductive success in salmonids (Dickerson et al., 2005; Garant, Dodson, & Bernatchez, 2001; Jones & Hutchings, 2002; Seamons, Bentzen, & Quinn, 2004).

The second well‐known morphological trait under selection is exaggerating secondary sexual characteristics of male salmonids, such as elongation of the jaw, development of an angular tip or kype, or dorsal hump, which is referred even in *the Descent of Man* (Darwin, 1871). Despite the wide acceptance of salmonids’ researchers, surprisingly few studies have actually demonstrated the roles of the sexual traits on reproductive success (Fleming & Gross, 1994). These exaggerated traits are usually observed in large sea‐run species or individuals, but even relatively small stream‐resident brook trout show such traits (Kazyak, Hilderbrand, & Holloway, 2013), providing a good opportunity to assess their roles. In fact, PC1 clearly distinguished between brook trout and white‐spotted charr based on secondary sexual characteristics. Thus, the significant positive effect of PC1 represents one of the rare instances of evidence for the role of male secondary sexual characteristics on reproductive success in the field.

The caudal peduncle area is also reported as an important factor for females in digging during nest construction and appears to influence the depth of egg burial (Fleming & Gross, 1994). By contrast, we did not find a relationship between caudal peduncle depth and reproductive success. This may be because our study occurred in an area where the substrate had been modified by humans and consisted of a concrete base with a shallow layer of pebbles, which likely reduced the available habitat variation in terms of potential nest depth. Based on a questionnaire to farmers, the channel modification occurred about 2002 (S. Fukui, personal communication), in which the time scale is too short for the trait evolution.

### 4.2 Reproductive success of hybrids

Our results clearly showed that both male and female hybrids have lower reproductive success compared to the parent species (Figure 3). This should be partly due to the maladaptive morphology as discussed above, but other undermined factors also reduce the reproductive success of hybrids because hybrid index had significant effects in both sexes. Unfortunately, the processes underlying the reduced hybrid reproductive success in our study are unknown, because our estimate of reproductive success represents a function of selection at different life stages, including spawning, eyed egg, egg‐to‐fry emergence and juvenile survival: selection could have acted at any or all of these stages. Previous laboratory studies have documented lower fitness of hybrids between BT and WSC at multiple stages (Suzuki & Fukuda, 1971, 1973, 1974). First, gonadal development of F1 hybrid males was reduced compared to the parental species (with lower quantity of milt) in both reciprocal crosses. Second, survival and growth rates at the early life stages (i.e., hatching and eyed eggs) of both F2 and some backcrossed hybrids were lower than those of parental species, although some hybrid vigor was also observed in F1 hybrids. Future studies should determine the relative reduction in hybrid fitness at each of the stages.

### 4.3 Conservation implications

We showed that reproductive success of male BT (and its hybrid) was reduced by the maladaptive phenotypes derived from WSC. This, in turn, suggests that native WSC may suffer from asymmetric introgression from non‐native BT, which have more remarkable secondary sexual characteristics. That is, hybrid WSC with more pronounced secondary sexual characteristics (via introgression with BT) might enjoy higher reproductive success compared to pure WSC. If this is the case, some genes of the non‐native species will persist and spread even when pure BT disappear (Fukui et al., 2016), which potentially reduces the likelihood of persistence of pure WSC. Although we could not directly assess the effects of introgression on the reproductive success of WSC due to insufficient sample size, our previous broader‐scale survey showed that most hybrids were derived from backcross with white‐spotted charr (Fukui et al., 2016). Future study should focus on the direction of hybridization and its mechanisms.

Reduced hybrid reproductive success may inhibit the spread of non‐native alleles and the development of hybrid swarms. We used, however, only 13 microsatellite markers to assess the levels of introgression, and therefore, non‐native (BT) alleles at other loci, particularly those that might be advantageous, may have spread more widely across the WSC genome. For instance, Fitzpatrick et al. (2010) documented the spread of a small portion of introduced alleles up to 90 km into native populations (the California Tiger Salamander: *Ambystoma californiense*) that have no record of non‐native species within 60 years, although a majority of single‐nucleotide polymorphism markers (65 of 68) showed no evidence of the spread beyond the region where non‐native species had been introduced. Further analysis using additional neutral and adaptive loci could improve our understanding of the risk in the spread of non‐native genes into WSC populations.

### 4.4 Limitations of the study

While genomic data from next‐generation sequencing (NGS) has recently been accumulating, we used only 13 microsatellite loci in the present study. Consequently, the hybrid index should be less accurate compared to NGS data. Also, we cannot know how much introgression has been occurring in the native species, as well as the loci under selection. In addition, parents were not determined for 40%–50% of juveniles, indicating that we could have missed some mature adults. We tried to collect most of the adults in the study reaches by 2–4 passes of electrofishing until few fish were captured in the last pass. However, since breeding dispersal is common in stream salmonids (Northcote, 1997; Hutchings and Gerber, 2002), some individuals might have moved to and reproduced in the study tributary between our sampling periods. Importantly, mainstem migratory fish often grow bigger (Koizumi, Yamamoto, & Maekawa, 2006) and, therefore, may attain higher reproductive success compared to smaller residents. We might have failed to collect such large migratory adults, which could explain the low parentage assignment. However, this is the nature of field studies in salmonids: similar attempts for estimating reproductive success showed the equivalent levels of parentage assignments (32%–61%: Thériault et al., 2007; Muhlfeld et al., 2009; Serbezov, Bernatchez, Olsen, & Vøllestad, 2010). Most importantly, even with the limited sampling, the results showed consistent patterns predicted from previous studies. Another important aspect, we missed is annual variation. A more detailed picture will be clarified with an NGS approach and additional surveys.

## 5 CONCLUSION

This study showed the relative reproductive success of hybrids and their parental species in the wild, despite that there are few field studies on directly quantifying relative fitness of hybrids and their parental species in human‐mediated hybrid zones. The reproductive success of hybrids was lower than that of the two parental species. Our results supported that both natural selection derived from outbreeding depression and sexual selection can affect the decision of hybrid fitness, and suggest that the more adaptive male traits derived from non‐native species may facilitate the genetic extinction of native population via asymmetric introgression. As this study could not completely separate effects of sexual selection from other factors which can influence hybrid fitness, to quantify other fitness components, such as survival or growth, would appear to be an effective approach for unraveling further causes reducing the hybrid fitness.

## CONFLICT OF INTEREST

None declared.

## AUTHOR CONTRIBUTIONS

SF and IK designed the study. SF and SLM conducted the field survey. SF performed DNA experiments and statistical analysis, and wrote the original manuscript. All authors contributed to the revisions of the manuscript.

## DATA ACCESSIBILITY

The data have been archived in Mendeley (https://data.mendeley.com/datasets/ss876j2wkz/1).
