# Supplementary material for: Maladaptive secondary sexual characteristics reduce the reproductive success of hybrids between native and non‐native salmonids
Source: Ecol Evol. 2018 Nov 14;8(23):12173–82. doi: 10.1002/ece3.4676 (PMC6303740; doi:10.1002/ece3.4676)
Supplement: Supplementary file 1 [file ECE3-8-12173-s001.docx]

**Supplemental information for:**

**Maladaptive secondary sexual characteristics reduce the reproductive success of hybrids between native and nonnative salmonids**

**Authors:** Sho Fukui^1^, Shannan L. May-McNally^2^, Eric B. Taylor^2^, Itsuro Koizumi^1,3^

^1^ Graduate School of Environmental Earth Science, Hokkaido University, N10W5 Sapporo, Hokkaido 060-0810, Japan

^2^ Department of Zoology, Biodiversity Research Centre and Beaty Biodiversity Museum, University of British Columbia, 6270 University Ave., Vancouver, BC, Canada

^3^ Faculty of Environmental Earth Science, Hokkaido University, N10W5 Sapporo, Hokkaido 060-0810, Japan

**Corresponding author**- Sho Fukui

**Address:** Graduate School of Environmental Earth Science, Hokkaido University, N10W5 Sapporo, Hokkaido 060-0810, Japan

**Email:** s-fukui@ees.hokudai.ac.jp

**Keywords:** adaptive introgression, invasive introgression, extrinsic factor, postzygotic isolation, sexual selection, invasive species

**Running title:** sexual selection against hybrid males


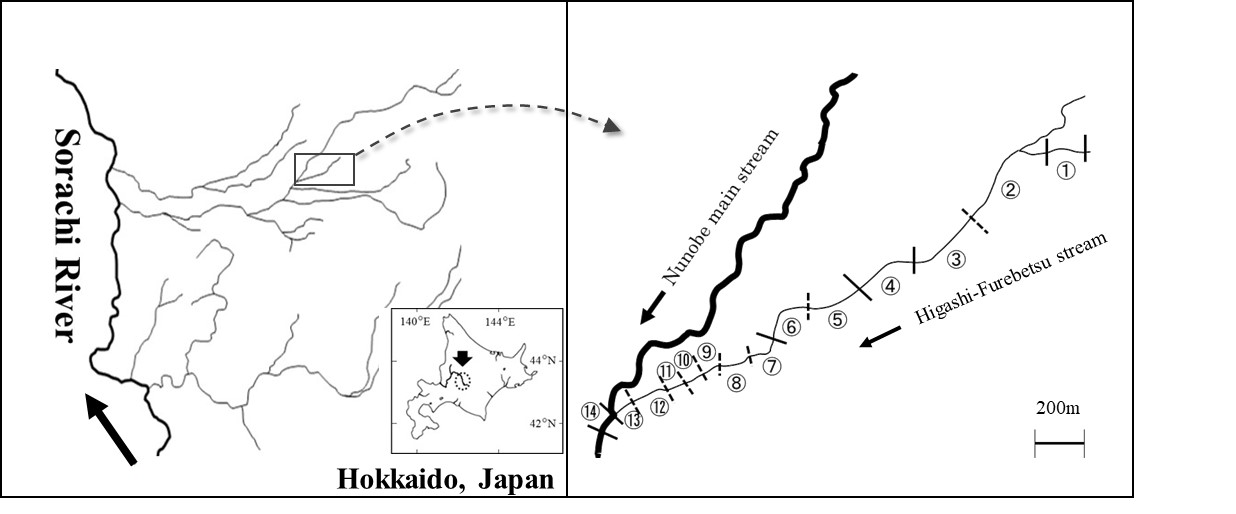


**Figure S1.** Location of study stream (Higashi-Furebetsu stream) in central Hokkaido, Japan. We established 14 study sections in the study　stream. Dashed lines represent dams which have approximately 1m height, respectively, in the study area. Solid lines indicate the boundaries without dam among the study sections.


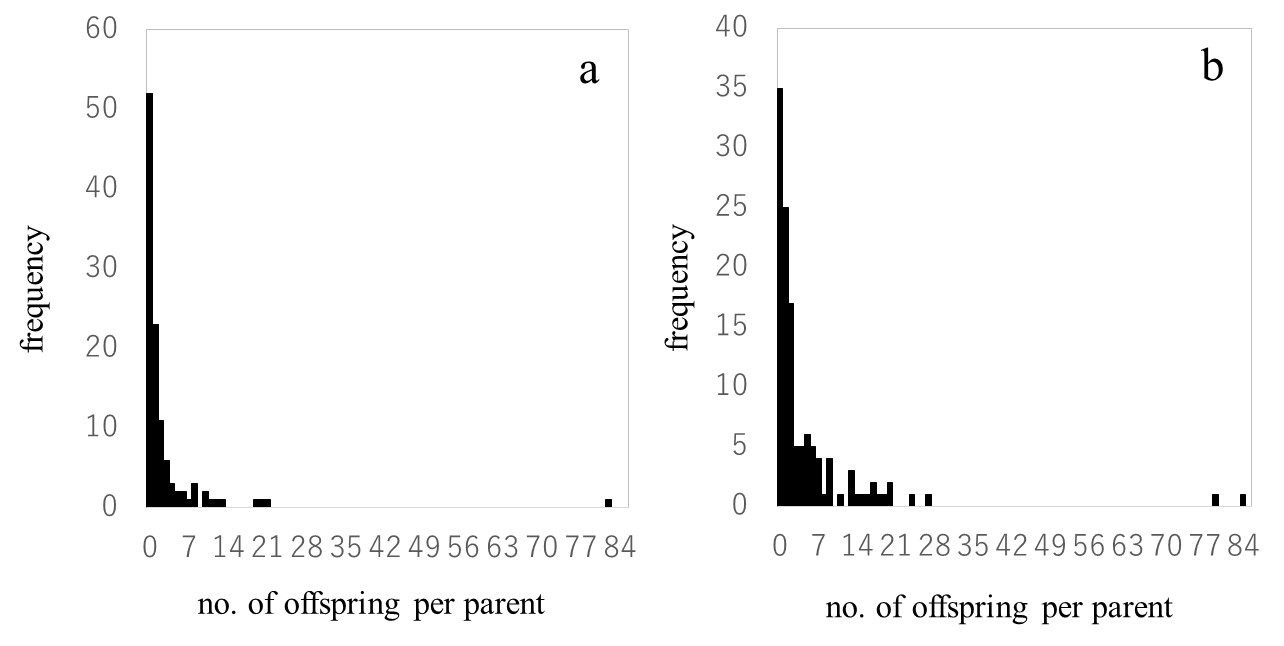


**Figure S2.** Frequency of assigned offspring in Higashi-Furebetsu stream for male (a) and female (b) brook trout, white-spotted charr, and their hybrids.

**Table S1.** Characteristics of the microsatellite loci used for parentage analysis of introduced brook trout, native white-spotted charr, and their hybrids in the Higashi-Furebetsu stream.

|  |  |  |  |  |  |  |  |  |  |  |  |  |  |  |  |  |  |  |  |  |
| --- | --- | --- | --- | --- | --- | --- | --- | --- | --- | --- | --- | --- | --- | --- | --- | --- | --- | --- | --- | --- |
|  | *Locus* |  | *Reference* |  | *Conc. (µM)* |  | *T_A_ (ºC)* |  | *Alleles* |  | *He* |  | *Pe (first parent)* |  | *Pe (identity)* |  | *BT size range* |  | *WSC size range* |  |
|  | *Multiplex 1* |  |  |  |  |  |  |  |  |  |  |  |  |  |  |  |  |  |  |  |
|  | Sco200 |  | Dehaan and Ardren 2005 |  | 0.17 |  | 56.0 |  | 24 |  | 0.4920 |  | 0.1283 |  | 0.6878 |  | 112-158 |  | 140-236 |  |
|  | Sco211 |  | Dehaan and Ardren 2005 |  | 0.17 |  | 56.0 |  | 19 |  | 0.5830 |  | 0.1838 |  | 0.7608 |  | 228-272 |  | 256-300 |  |
|  | Sle6 |  | Yamaguchi et al. 2008 |  | 0.33 |  | 56.0 |  | 8 |  | 0.5240 |  | 0.1388 |  | 0.6790 |  | 280-282 |  | 282-290 |  |
|  | Ssa197** |  | O'Reilly et al. 1996 |  | 0.17 |  | 56.0 |  | 5 |  | 0.1520 |  | 0.0117 |  | 0.2756 |  | 148 |  | 115-120 |  |
|  | SsosL456* |  | Slettan et al. 1997 |  | 0.08 |  | 56.0 |  | 2 |  | 0.1210 |  | 0.0073 |  | 0.2201 |  | 156 |  | 160 |  |
|  | *Multiplex 2* |  |  |  |  |  |  |  |  |  |  |  |  |  |  |  |  |  |  |  |
|  | Smm21* |  | Crane et al. 2004 |  | 0.44 |  | 56.0 |  | 4 |  | 0.1540 |  | 0.0119 |  | 0.2752 |  | 144 |  | 114 |  |
|  | Otsg83b* |  | Williamson et al. 2002 |  | 0.22 |  | 56.0 |  | 16 |  | 0.2970 |  | 0.0448 |  | 0.2458 |  | 75-79 |  | 160-226 |  |
|  | *Multiplex 3* |  |  |  |  |  |  |  |  |  |  |  |  |  |  |  |  |  |  |  |
|  | Mst85** |  | Presa and Guyomard 1996 |  | 0.07 |  | 56.0 |  | 5 |  | 0.5040 |  | 0.1274 |  | 0.6807 |  | 154-166 |  | 120 |  |
|  | Sfo12** |  | Angers et al. 1995 |  | 0.29 |  | 56.0 |  | 11 |  | 0.2900 |  | 0.0438 |  | 0.4810 |  | 270-272 |  | 208-240 |  |
|  | Otsg253b* |  | Williamson et al. 2002 |  | 0.07 |  | 56.0 |  | 10 |  | 0.6140 |  | 0.2058 |  | 0.7870 |  | 136-160 |  | 84-88 |  |
|  | Sco216 |  | Dehaan and Ardren 2005 |  | 0.21 |  | 56.0 |  | 3 |  | 0.1510 |  | 0.0113 |  | 0.2673 |  | 141-153 |  | 141 |  |
|  | Sco220 |  | Dehaan and Ardren 2005 |  | 0.29 |  | 56.0 |  | 28 |  | 0.7630 |  | 0.3867 |  | 0.3867 |  | 264-312 |  | 280-380 |  |
|  | Omm1105 |  | Rexroad et al. 2002 |  | 0.14 |  | 56.0 |  | 23 |  | 0.7470 |  | 0.3636 |  | 0.9048 |  | 122-152 |  | 134-206 |  |

Conc., final PCR concentration of forward and reverse primers; T_A_, annealing temperature; Alleles, total number of observed alleles; H_E_, expected heterozygosity; P_E_ (first parent), exclusion probability for the first parent; P_E_ (identity), average probability that the set of loci will fail to differentiate between two randomly-selected individuals; Combined exclusion probabilities were 0.8549 and >0.9999 for P_E_ (first parent) and P_E_ (identity), respectively. Calculations were made using CERVUS 3.0.7. The size range and of alleles observed at each locus for each species which has >99.9% of own genetic admixture is given. ** Loci are diagnostic between white-spotted charr and brook trout described by previous studies, and * loci shows diagnostic between white-spotted charr and brook trout indicated by the present study.

**References**

Angers, B., Bernatchez, L., Angers, A., Desgroseillers, L. (1995). Specific microsatellite loci for brook charr reveal strong population subdivision on a microgeographic scale. Journal of Fish Biology, 47, 177–185.

Crane, P. A., Lewis, C. J., Kretschmer, S. J., Miller, S. J., Spearman, W. J., DeCicco, A. L., & Wenburg, J. K. (2004). Characterization and inheritance of seven microsatellite loci from Dolly Varden, Salvelinus malma, and cross-species amplification in Arctic Char, S. alpinus. Conservation Genetics, 5, 737–741.

Dehaan, P. W. & Ardren, W. R. (2005). Characterization of 20 highly variable tetranucleotide microsatellite loci for bull trout (Salvelinus confluentus) and cross-amplification in other Salvelinus species. Molecular Ecology Notes, 5, 582–585.

O’Reilly, P. T., Hamilton, L. C., McConnell, S. K., & Wright, J. M. (1996). Rapid analysis of genetic variation in Atlantic salmon (Salmo salar) by PCR multiplexing of dinucleotide and tetranucleotide microsatellite. Canadian. Journal of Fisheries and Aquatic Sciences, 53, 2292–2298.

Presa, P., Guyomard, R. (1996). Conservation of microsatellites in three species of salmonids. Journal of Fish Biology, 49, 1326–1329.

Rexroad, C. E., Coleman, R. L., Hershberger, W. K., & Killefer, J. (2002). Rapid communication: thirty-eight polymorphic microsatellite markers for mapping in rainbow trout. Journal of Animal Science, 80, 541–542.

Slettan, A., Olsaker, I. & Oystein, L. (1997). Segregation studies and linkage analysis of Atlantic salmon microsatellites using haploid genetics. Heredity, 78, 620–627.

Williamson, K., S., Cordes, J. F., & May, B. P. (2002). Characterization of microsatellite loci in Chinook salmon (Oncorhynchus tshawytscha) and cross-species amplification in other salmonids. Molecular Ecology Notes, 2, 17–19.

Yamaguchi, K., Nakajima, M., & Taniguchi, N. (2008). Development of microsatellite markers in Japanese char Salvelinus leucomaenis and their applicability to closely related species. Fish Genetics and Breeding Science, 38, 123–130.
